# Biofortified Diets Containing Algae and Selenised Yeast: Effects on Growth Performance, Nutrient Utilization, and Tissue Composition of Gilthead Seabream (*Sparus aurata*)

**DOI:** 10.3389/fphys.2021.812884

**Published:** 2022-01-13

**Authors:** Mariana Ferreira, Pedro C. Ribeiro, Laura Ribeiro, Marisa Barata, Valentina F. Domingues, Sara Sousa, Cristina Soares, Alexandra Marques, Pedro Pousão-Ferreira, Jorge Dias, L. Filipe C. Castro, António Marques, Maria L. Nunes, Luisa M. P. Valente

**Affiliations:** ^1^Centro Interdisciplinar de Investigação Marinha e Ambiental (CIIMAR), Universidade do Porto, Matosinhos, Portugal; ^2^Instituto de Ciências Biomédicas Abel Salazar (ICBAS), Universidade do Porto, Porto, Portugal; ^3^Departamento de Biologia, Faculdade de Ciências (FCUP), Universidade do Porto, Porto, Portugal; ^4^Estação Piloto de Piscicultura de Olhão, Instituto Português do Mar e da Atmosfera (EPPO-IPMA), Olhão, Portugal; ^5^REQUIMTE/LAQV, Instituto Superior de Engenharia do Porto, Instituto Politécnico do Porto, Porto, Portugal; ^6^Divisão de Aquacultura, Valorização e Bioprospecção, Instituto Português do Mar e da Atmosfera (DivAV-IPMA), Lisbon, Portugal; ^7^Sparos Lda., Olhão, Portugal

**Keywords:** iodine, *Laminaria digitata*, microalgae, omega-3 fatty acids, selenium

## Abstract

Efforts have been made to find natural, highly nutritious alternatives to replace fish meal (FM) and fish oil (FO), which can simultaneously promote fish health and improve the nutritional quality of filets for human consumption. This study evaluated the impact of biofortified diets containing microalgae (as replacement for FM and FO), macroalgae (as natural source of iodine) and selenised yeast (organic source of selenium) on gilthead seabream growth, nutrient utilization, tissue composition and gene expression. A control diet (CTRL) with 15% FM and 5.5% FO was compared with three experimental diets (AD1, AD2, and AD3), where a microalgae blend (*Chlorella* sp., *Tetraselmis* sp., and DHA-rich *Schizochytrium* sp.) replaced 33% of FM. Diet AD1 contained 20% less FO. Diets were supplemented with *Laminaria digitata* (0.4% AD1 and AD2; 0.8% AD3) and selenised yeast (0.02% AD1 and AD2; 0.04% AD3). After feeding the experimental diets for 12 weeks, growth was similar in fish fed AD1, AD2, and CTRL, indicating that microalgae meal can partially replace both FM and FO in diets for seabream. But AD3 suppressed fish growth, suggesting that *L. digitata* and selenised yeast supplementation should be kept under 0.8 and 0.04%, respectively. Despite lower lipid intake and decreased PUFAs bioavailability in fish fed AD3, compared to CTRL, hepatic *elovl5* was upregulated resulting in a significant increase of muscle EPA + DHA. Indeed, filets of fish fed AD2 and AD3 provided the highest EPA + DHA contents (0.7 g 100 g^–1^), that are well above the minimum recommended values for human consumption. Fish consuming the AD diets had a higher retention and gain of selenium, while iodine gain remained similar among diets. Upregulation of selenoproteins (*gpx1*, *selk*, and *dio2*) was observed in liver of fish fed AD1, but diets had limited impact on fish antioxidant status. Overall, results indicate that the tested microalgae are good sources of protein and lipids, with their LC-PUFAs being effectively accumulated in seabream muscle. Selenised yeast is a good fortification vehicle to increase selenium levels in fish, but efforts should be placed to find new strategies to fortify fish in iodine.

## Introduction

Fish are major dietary sources of high-quality proteins, vitamins (i.e., D, A and B12), n-3 long-chain polyunsaturated fatty acids (n-3 LC-PUFAs), mainly EPA (eicosapentaenoic acid) and DHA (docosahexaenoic acid), iodine, selenium and iron for human consumption (EFSA, 2014), so farmed products must also provide adequate levels of such nutrients for consumers.

Gilthead seabream (*Sparus aurata*) is one of the main marine fish species for aquaculture production in the Mediterranean region (FAO, 2020). Plant-based products are now commonly used as substitutes for fish meal (FM) and fish oil (FO) in diets for several carnivorous farmed fish, but previous studies have shown that complete, or even partial, replacement of FM and FO with plant proteins (PP) and vegetable oils (VOs) in diets for *S. aurata* can compromise fish health and nutritional value of filets for human consumption (Izquierdo et al., 2005; Montero et al., 2008; Matos et al., 2017; Estruch et al., 2018). In this context, diets for farmed fish using natural products, such as selected microalgae (rich in nutrients including LC-PUFAs and bioactive compounds), seaweeds (rich in iodine) and selenised yeast (rich in selenium), with proven beneficial effects for both fish performance and filet quality, are of interest to both the scientific community and aquaculture sector (Vizcaíno et al., 2016; Ribeiro et al., 2017; Wang et al., 2018; Granby et al., 2020; Valente et al., 2021). However, fortification efficiency for a given nutrient is a complex process depending on many factors including origin, dose, form, and potential interactions with other dietary components (Ramos et al., 2008; Ribeiro et al., 2017; Saffari et al., 2017). Hence, new fortification strategies are required to maximize deposition of beneficial compounds contributing to the functional foods market in parallel with securing fish health.

The use of microalgae in fish diets has been explored previously, as promising sources of nutrients and bioactive compounds (Valente et al., 2021). Microalgae have been used successfully as partial replacements for FM and FO in diets for marine fish (Tibaldi et al., 2015; Cardinaletti et al., 2018; Batista et al., 2020). In European seabass (*Dicentrarchus labrax*), growth was not affected by the replacement of (45%) protein and (36%) lipids from FM with a blend of *Tisochrysis lutea* and *Tetraselmis suecica* (Cardinaletti et al., 2018). Seong et al. (2021) observed good results in terms of growth performance with the inclusion of mixed microalgae (*Schizochytrium* sp., *Nannochloropsis* sp. and *Chlorella* sp.) in non-FM and non-FO diets for red sea bream (*Pagrus major*), at an inclusion level of 45%. Thus, *Tetraselmis* sp. and *Chlorella* sp., due to their high protein content (35–60% dry weight), are potential candidates for inclusion in aquafeeds (Acquah et al., 2020). Certain microalgae, specifically *Schizochytrium* sp., are also rich sources of lipids and n-3 LC-PUFAs, such as docosahexaenoic acid (DHA) (Tocher, 2015), which together with eicosapentaenoic acid (EPA) have a crucial physiological role in both humans and fish (Swanson et al., 2012). In gilthead seabream larvae, dietary substitution of FO with *Schizochytrium* sp. resulted in whole-body DHA concentrations similar to those in fish fed FO-only diets (Ganuza et al., 2008). Like many other marine fish, the *S. aurata* capacity to synthesize highly unsaturated fatty acids (HUFAs) is limited, due to its low Δ5 desaturase activity (Tocher, 2010, 2015). However, several studies have shown that genes involved in synthesis of these fatty acids, namely desaturases and elongases, can be modulated nutritionally (Izquierdo et al., 2008; Benedito-Palos et al., 2014; Houston et al., 2017).

Macroalgae are also rich sources of bioactive compounds and their inclusion in aquafeeds has been associated with beneficial effects, including improved fish growth, welfare, and nutritional content of filets (Dantagnan et al., 2009; Wassef et al., 2013; Zeynali et al., 2020). Iodine-rich brown algae, such as *Saccharina latissima* and *Laminaria digitata*, have been shown to be promising vehicles for fortifying filets with iodine (Ribeiro et al., 2015, 2017; Granby et al., 2020). But previous reports have also evidenced impacts on lipid metabolism among several fish species, as a result of including macroalgae in diets (Xuan et al., 2013; Morshedi et al., 2018). In rainbow trout, for example, the dietary inclusion of *S. latissima* led to a downregulation of hepatic fatty acid synthase (*fas*) and a general reduction in fatty acid filet content. Nevertheless, EPA and DHA filet content did not decrease with inclusion of macroalgae, indicating a selective retention of these essential fatty acids in the muscle (Ferreira et al., 2020). Both micro- and macroalgae are also natural sources of compounds with bioactive properties, specifically carotenoids and polyphenols, capable of improving fish antioxidant status (Magnoni et al., 2017; Peixoto et al., 2019; Teimouri et al., 2019).

Selenium is an essential micronutrient involved in several biological processes in fish. Evidence suggests that organic Se has a higher bioavailability than inorganic forms (Khan et al., 2017; Sele et al., 2018) and is more easily incorporated into selenoproteins, involved in thyroid hormones metabolism (deiodinases) and oxidative stress pathways (glutathione peroxidase) (Mariotti et al., 2012). Dietary supplementation with selenised yeast was associated with increased growth of rainbow trout (Ribeiro et al., 2017; Wang et al., 2018) and *Megalobrama amblycephala* (Liu et al., 2017). Wang et al. (2018) also reported upregulation of several selenoproteins, both in liver and muscle, which were positively correlated with growth. In gilthead seabream, little is known about the impact of dietary selenised yeast or other organic forms of selenium on fish growth and metabolism (Mechlaoui et al., 2019).

This study aimed to evaluate the impact of biofortified diets containing a blend of microalgae (*Chlorella* sp., *Tetraselmis* sp., and DHA-rich *Schizochytrium* sp.), as partial replacement of FM alone or FM and FO. These diets were also supplemented with a seaweed, *L. digitata* (0.4 and 0.8%), and selenised yeast (0.02 and 0.04%), as natural sources of iodine and selenium, respectively. The effects of these biofortification strategies on growth performance, nutrient utilization and filet composition were assessed. Expression of genes involved in growth, selenium and iodine metabolism, oxidative stress, and lipid metabolism pathways were also evaluated to provide further insights into the potential of natural products (algae and yeast) to modulate fish growth and metabolism, nutrient utilization, and filet nutritional value for human consumption.

## Materials and Methods

### Ethics Statement

Fish trials were conducted in compliance with the guidelines of the European Union (Directive 2010/63/EU) and approved by the Ethical Committee of the EPPO-IPMA (Estação Piloto de Piscicultura de Olhão, Av. Parque Natural da Ria Formosa N, 8700-194, Olhão), overseen by the National Competence Authority.

### Experimental Diets

Gilthead seabream were fed with four isonitrogenous (50% dry matter, DM) and isoenergetic (24 kJ g^–1^ DM) diets, formulated and extruded by Sparos Lda., Olhão, Portugal. Feed ingredients and diet compositions are summarized in Table 1: a control diet (CTRL) with moderate FM (15%) and FO (5.5%) contents was compared with three experimental diets (AD1, AD2. and AD3) with 33% FM replaced with microalgae meal (*Chlorella* sp., *Tetraselmis* sp. and DHA-rich *Schizochytrium* sp.) and reduced vegetable oil contents (11% reduction in soybean, rapeseed, and linseed in AD1 and between 21–23% reduction in AD2 and AD3). AD1 also contained 20% less FO. The CTRL diet contained 1.34 mg kg^–^^1^ of iodine and 0.76 mg kg^–1^ of selenium. All AD diets were enriched with both iodine-rich macroalgae (*L. digitata*) and selenised yeast: AD1 and AD2 contained the same amount of *L. digitata* and selenised yeast (0.4 and 0.02%, respectively), providing iodine levels at 40% maximum legal limit of 20 mg kg^–1^ (8.10 and 8.97 mg kg^–1^ DM, respectively); and selenium levels at 150% of CTRL diet (1.15 and 1.07 mg kg^–1^ DM, respectively); whereas AD3 was supplemented with 0.8% *L. digitata* and 0.04% selenised yeast, providing iodine levels at 70% maximum legal limit (14.59 mg kg^–1^ DM) and selenium levels at 185% of CTRL diet (1.41 mg kg^–1^ DM). The adoption of selenium levels above the maximum legal limit is justified by the interest to assess any potential beneficial and/or detrimental effects of this trace mineral in the context of a biofortification feeding strategy.

**TABLE 1:** Ingredients and proximate composition of the experimental diets.

Ingredients (%)	CTRL	AD1	AD2	AD3
Fishmeal LT70^1^	15.0	10.0	10.0	10.0
Fish protein concentrate^2^	2.5	2.5	2.5	2.5
Porcine blood meal^3^	2.5	2.5	2.5	2.5
Microalgae meal (*Chlorella* sp.)^4^		5.0	5.0	5.0
Microalgae meal (*Tetraselmis* sp.)^5^		0.5	0.5	0.5
Microalgae meal (*Schizochytrium* sp.)^6^		3.2	3.2	3.2
Macroalgae meal (*Laminaria digitata*)^7^		0.4	0.4	0.8
Soy protein concentrate^8^	17.0	17.0	17.0	17.0
Wheat gluten^9^	12.0	12.0	12.0	12.0
Corn gluten meal^10^	8.0	8.0	8.0	8.0
Soybean meal 48^11^	8.0	8.0	8.0	8.0
Wheat meal^12^	16.6	13.9	13.9	13.5
Fish oil^13^	5.5	4.4	5.5	5.5
Soybean oil^14^	2.8	2.5	2.2	2.12
Rapeseed oil^14^	5.6	5.0	4.3	4.3
Linseed oil^14^	0.9	0.8	0.7	0.7
Vitamins and minerals premix^15^	1.1	1.1	1.1	1.1
Binder^16^	1.0	1.0	1.0	1.0
Antioxidant^17^	0.2	0.2	0.2	0.2
Sodium propionate ^18^	0.1	0.1	0.1	0.1
Monoammonium phosphate^19^	0.5	1.0	1.0	1.0
Selenised yeast^20^		0.02	0.02	0.04
L-Tryptophan ^21^	0.1	0.1	0.1	0.1
D,L-Methionine^22^	0.2	0.3	0.3	0.3
L-Taurine^23^	0.4	0.5	0.5	0.5
**Proximate composition**				
Dry matter (DM,%)	92.1	91.1	90.9	91.2
Crude protein (% DM)	50.5	50.5	50.7	50.4
Crude fat (% DM)	16.7	16.2	15.7	14.8
Ash (% DM)	5.7	5.7	6.0	5.7
Energy (kJ g^–1^ DM)	23.8	24.0	23.9	23.8
Iodine (mg kg^–1^ DM)	1.34	8.10	8.97	14.59
Selenium (mg kg^–1^ DM)	0.76	1.15	1.07	1.41

*^1^NORVIK LT: 70.6% crude protein (CP), 5.8% crude fat (CF), Sopropêche, France;*

*^2^ CPSP90: 86% CP, 6% CF, Sopropêche, France;*

*^3^Porcine blood meal: 89% CP, 1% CF, SONAC BV, Netherlands;*

*^4^Chlorella sp. meal: 62% CP, 9% CF, ALLMICROALGAE, Portugal;*

*^5^Tetraselmis sp. meal: 23% CP, 6.2% CF, ALLMICROALGAE, Portugal;*

*^6^ALL-G RICH Schizochytrium sp.: 9.8% CP, 63.1% CF, Alltech Portugal;*

*^7^Dry Laminaria digitata: 5.4% CP, 0.5% CF, 3700 mg iodine kg^–1^, Agrimer, France;*

*^8^Soycomil P: 6 3% CP, 0.8% CF, ADM, Netherlands;*

*^9^VITEN: 82% CP, 2.1% CF, Roquette, France;*

*^10^Corn gluten meal: 61% CP, 6% CF, COPAM, Portugal;*

*^11^Solvent extracted dehulled soybean meal: 47% CP, 2.6% CF, CARGILL, Spain;*

*^12^Wheat meal: 10.2% CP, 1.2% CF, Casa Lanchinha, Portugal;*

*^13^Sopropêche, France;*

*^14^Henry Lamotte Oils GmbH, Germany;*

*^15^INVIVONSA Portugal SA, Portugal: Vitamins (IU or mg kg^–1^ diet): DL-alpha tocopherol acetate, 100 mg; sodium menadione bisulfate, 25 mg; retinyl acetate, 20000 IU; DL-cholecalciferol, 2000 IU; thiamin, 30 mg; riboflavin, 30 mg; pyridoxine, 20 mg; cyanocobalamin, 0.1 mg; nicotinic acid, 200 mg; folic acid, 15 mg; ascorbic acid, 500 mg; inositol, 500 mg; biotin, 3 mg; calcium pantothenate, 100 mg; choline chloride, 1000 mg, betaine, 500 mg. Minerals (g or mg kg^–1^ diet): copper sulfate, 9 mg; ferric sulfate, 6 mg; potassium iodide, 0.5 mg; manganese oxide, 9.6 mg; sodium selenite, 0.01 mg; zinc sulfate, 7.5 mg; sodium chloride, 400 mg; excipient wheat middling’s;*

*^16^Guar gum, SEAH International, France;*

*^17^VERDILOX, Kemin Europe NV, Belgium;*

*^18^PREMIX LDA., Portugal;*

*^19^Windmill AQUAPHOS, 26% P, ALIPHOS, Belgium;*

*^20^ALKOSEL R397: 2200 mg selenium kg^–1^, Lallemand, France;*

*^21^TrypAMINO 98%, Evonik Nutrition & Care GmbH, Germany;*

*^22^DL-Methionine for Aquaculture 99%, EVONIK Nutrition & Care GmbH, Germany;*

*^23^L-Taurine 98%, ORFFA, Netherlands.*

All formulated diets were isonitrogeneous and isoenergetic (Table 1). Moreover, as expected AD1 and AD2 had similar concentrations of iodine and selenium, that were 6- and 1.5-times higher than the CTRL, respectively. AD3 had 86% more Se and an iodine concentration that was 11-times higher than the CTRL diet. The fatty acid composition (% total fatty acids) of the diets is presented in Table 2. The CTRL diet had the lowest concentrations of saturated fatty acids (SFAs), primarily due to the low palmitic acid (16:0) content; the highest concentrations of monounsaturated fatty acids (MUFAs), as a result of the highest oleic acid (OA, 18:1 n-9 c) content; and the highest concentrations of polyunsaturated fatty acids (PUFAs) due to the highest linoleic acid (LA, 18:2 n-6 c) and eicosapentaenoic acid (EPA, 20:5 n-3) contents. AD2 and AD3 had similar fatty acids contents with the highest concentrations of SFAs and lowest MUFAs. Compared to CTRL, all AD diets had higher docosahexaenoic acid (DHA, 22:6 n-3) contents (5–6 vs. 3% total fatty acids), resulting in higher DHA:EPA ratios (1.2–1.4 vs. 0.7). AD1, which had an additional 20% reduction in FO, also had the highest DHA:EPA ratio due to the lowest EPA content (3.8 vs. 4.5–4.7% total fatty acids in all other diets). Within the AD diets, AD1 had the lowest contents of SFAs and the lowest concentration of DHA (5.3 vs. 5.7–5.8% total fatty acids).

**TABLE 2:** Fatty acid composition of the experimental diets (% total fatty acids).

Fatty acids	CTRL	AD1	AD2	AD3
14:0	2.52	2.65	3.23	3.26
16:0	12.25	16.94	18.53	18.54
18:0	2.99	2.75	3.05	3.07
^1^Σ SFA	19.42	24.41	27.06	27.11
16:1 n-7	2.76	2.35	2.70	2.70
18:1 n-7	2.52	2.26	2.24	2.22
18:1 n-9 c (OA)	29.95	26.62	24.06	24.04
20:1 n-9	1.39	1.12	1.13	1.13
^2^Σ MUFA	38.72	34.41	32.31	32.25
18:2 n-6 c (LA)	21.41	19.96	18.28	18.34
18:3 n-3 (ALA)	6.66	6.39	5.81	5.88
20:5 n-3 (EPA)	4.69	3.84	4.56	4.53
22:6 n-3 (DHA)	3.41	5.27	5.80	5.74
*^a^*EPA + DHA	8.11	9.11	10.35	10.28
DHA/EPA	0.73	1.37	1.27	1.27
^3^Σ PUFA	39.79	39.13	38.66	38.66

*^1^Σ SFA is the sum of saturated fatty acids and also includes 15:0, 17:0, 20:0, 22:0 and 24:0;*

*^2^Σ MUFA is the sum of mono-unsaturated fatty acids and also includes 17:1 n-7, 22:1 n-9, 22:1 n-11 and 24:1 n-9;*

*^3^Σ PUFA is the sum of polyunsaturated fatty acids and also includes 18:3 n-6, 18:4 n-3, 20:2 n-6, 20:3 n-6, 20:4 n-6, 21:5 n-3, and 22:5 n-3;*

*^a^EPA + DHA = eicosapentaenoic acid + docosahexaenoic acid. OA: oleic acid; LA: linoleic acid; ALA: α-linolenic acid.*

### Experimental Trial and Fish Sampling

The trial was conducted in Olhão, Portugal, at the aquaculture research facility EPPO-IPMA between 9th July and 1st October 2018 (85 days). After quarantine (3 weeks), homogenous groups of fish (373.91 ± 10.16 g) were established and distributed into 12 identical circular fiberglass tanks (3 m^3^ tanks; initial fish density of 6 kg m^–3^) in an open seawater system. The system was supplied with continuous water flow (25.2 ± 1.4°C, 5.6 ± 0.9 mg L^–1^ dissolved oxygen). All the tanks were subjected to natural photoperiod summer conditions (14 light/10 dark). Experimental diets were tested in triplicate tanks (*n* = 50 fish per replicate/tank) and fish were hand-fed *ad libitum* four times a day, seven days a week. At the beginning and end of the trial, fish were bulk weighed and six fish per tank (18 fish per treatment) were anesthetized (phenoxyethanol, 500 ppm) before being weighed individually and their lengths recorded. These fish were euthanized by spinal cord section and stored at –20°C for proximate whole-body composition analysis. After 12 weeks, five fish per tank (15 fish per treatment) were anesthetized (phenoxyethanol, 500 ppm), measured and weighed, and euthanized for the collection of tissue samples. These fish were dissected, and viscera, liver and muscle weighed; livers and muscle were collected for further analysis. For muscle analysis, skinless fish fillets were collected and pooled (five fish fillets per pooled sample), resulting in three pooled samples per dietary treatment. For gene expression, small portions of liver tissue (five fish per tank) were collected from each fish and preserved immediately in RNAlater™ (Thermo Fisher Scientific, Waltham, United States). Then, the samples were transferred to 4°C for 24 h before being stored at –80°C until RNA extraction. For oxidative stress analysis, portions of liver from the same fish were stored at –80°C.

### Proximate Composition of Feed and Whole-Body

Fish collected from each tank (*n* = 6) were ground, pooled and freeze-dried for proximate composition analysis. Experimental diets were also homogenized prior to analysis. Proximate composition of whole-body samples and experimental diets were performed in duplicate, according to AOAC (2006) methods: dry matter (in an oven at 105°C to constant weight); ash (incinerated at 500°C for 5 h in a muffle furnace; Nabertherm L9/11/B170, Bremen, Germany); protein by quantitation of nitrogen (N) using a Leco nitrogen analyzer (Model FP-528, Leco Corporation, St. Joseph, United States) and conversion (N × 6.25) to equivalent protein; gross energy using an adiabatic bomb calorimeter (Werke C2000, IKA, Staufen, Germany). Total lipids were extracted and quantified from whole-body and muscle samples according to Folch et al. (1957) and using Folch solution (dichloromethane:methanol 2:1 v/v with 0.01% butylated hydroxytoluene – BHT). Iodine and selenium contents of feed samples were determined according to the European standards EN 15111:2007 and EN 15763:2009, respectively. Inductively coupled plasma mass spectrometer (ICP-MS) (Thermo X series II, Thermo Fisher Scientific, Waltham, United States), after alkaline (iodine) or acid (selenium) digestion, was used for determination of iodine and selenium, as described by Barbosa et al. (2020).

### Fatty Acid Analysis of Feed, Whole-Body, and Muscle

Fatty acid methyl esters (FAMEs) of muscle and whole-body lipid extracts and experimental diets were *trans*-esterified, according to Bondia-Pons et al. (2007). Samples were diluted in 1 mL of *n*-hexane and 100 μg L^–1^ of internal standard (13:0 or 21:0), and 5 mL of sodium methylate solution was added (0.5 M, NaOMe). After vigorous shaking, samples were heated to 100°C for 10 min and cooled for 5 min on ice. Samples were esterified with 5 mL of boron trifluoride-methanol at 100°C for 30 min and cooled to room temperature. FAMEs were isolated by adding 1 mL of *n*-hexane containing BHT (0.02%), the tubes were shaken, and 2 mL of sodium chloride (58 g L^–1^) was added. Following centrifugation for 10 min at 2,000 × *g*, the top layer was retrieved and dried with anhydrous sodium sulfate. An aliquot (150 μL) was evaporated to dryness under nitrogen and re-constituted in *n*-hexane (1100 μL) before 100 μL of this sample was placed in a glass vial with an insert and subjected to analysis using flame ionization detection (GC-FID). A Shimadzu GC-2010 gas chromatograph (Kyoto, Japan) equipped with a FID and a Shimadzu AOC-20i autoinjector was used. Separation was carried out on an Agilent J&W CP-Sil 88 capillary column (60 m × 0.25 mm I.D., film thickness 0.20 μm). Operating conditions were as follows: split mode, with split ratio of 1:50 and an injection volume of 1.5 μL. The injector and detector temperatures were kept at 250 and 260°C, respectively. A flow rate of 30 mL min^–1^ of helium as carrier gas (Linde Sógas purity ≥ 99.999%), 40 mL min^–1^ of hydrogen, and 400 mL min^–1^ of air was provided. The column thermal gradient was as follows: 100°C for 5 min, then raise to 215°C at 1°C min^–1^ and held at this temperature for 12 min. FAMEs were identified by comparison with a known standard mixture (Sigma 47,885-U Supelco 37 Component FAME Mix, Darmstadt, Germany) and quantified using the software GCsolution for GC systems (Shimadzu, Kyoto, Japan). FAME contents in muscle tissue were expressed on the % of the total FAME basis. Quantification of each FAME as mg g^–1^ sample was estimated using tridecanoic acid (13:0) or heneicosanoic acid (21:0) as internal standards.

### RNA Extraction and cDNA Synthesis

Total RNA was extracted from 15 mg of liver (*n* = 9 per treatment), using NZYol (Nyztech, Lisbon, Portugal) following the manufacturer’s instructions, with some modifications, as previously described by Ferreira et al. (2020). RNA quantity and purity (A260/A280 ratios) were evaluated by spectrophotometry, using a Take 3 micro-volume plate on a Synergy™ HT Multi-Detection Reader and Gen5™ software (BioTek Instruments, Winooski, VT, United States). RNA integrity was assessed in 1% TAE (w/v) agarose electrophoresis gel stained with GelRed™ (Biotium, Hayward, United States). Subsequently, cDNA was synthesized from 1 μg of total RNA using a NZY First-Strand cDNA Synthesis Kit (Nyztech, Lisbon, Portugal), following the standard protocol.

### Gene Expression Analysis

Reverse transcription (RT) q-PCR was performed to assess expression of 13 genes (Table 3) involved in (a) growth: *igf2*, insulin-like growth factor 2; (b) selenium metabolism: *selk*, selenoprotein K and *selp*, selenoprotein P; (c) iodine metabolism: *dio1*, iodothyronine deiodinase 1 and *dio2*, iodothyronine deiodinase 2; (d) oxidative stress: *gpx1a*, glutathione peroxidase 1a, *gpx1b*, glutathione peroxidase 1b, and *gpx4b*, glutathione peroxidase 4b; and (e) lipid metabolism: *cpt1a*, carnitine palmitoyl transferase 1a (lipolytic pathway related gene); *fas*, fatty acid synthase and *srebp1*, sterol regulatory element binding protein 1 (lipogenic pathway related genes); *fads2*, fatty acid desaturase 2 and *elovl5*, elongation of very long chain fatty acids protein 5 (desaturation and elongation pathway related genes). RT-qPCR was carried out using primers designed in previous studies (Table 3). For *selk*, *selp*, *dio1*, *dio2*, gene sequences were retrieved from NCBI GenBank and primers were designed with NCBI Primer Blast Tool (Ye et al., 2012), according to known qPCR restrictions: one of the primers was located at an exon-exon junction to avoid amplification of genomic DNA; primer length (18–22 bp); amplicon size (80–150 bp); Tm difference between primers; GC content (50–60%); and self-dimer or cross-dimer formation. PCR was performed in Eppendorf Mastercycler ep Gradient S (Eppendorf, Hamburg, Germany) using NZYSpeedy qPCR Green Master Mix (2x) (Nyztech, Lisbon, Portugal), according to Ferreira et al. (2020). Specific annealing temperatures and PCR efficiencies for the target genes can be found in Table 3. Samples and standard curves were run in duplicate as well as a negative control without cDNA. Stabilities of three candidate reference genes (β*-actin*, *ef1*α and *rps18*) were determined using the geNorm algorithm (Vandesompele et al., 2002) implemented in qbase + software, version 3.2 (Biogazelle, Zwijnaarde, Belgium^1^). Based on these stabilities, geometric means for *ef1*α, *rps18* and β*actin* was used to calculate normalization factors for each liver sample. To evaluate transcript levels, 2^–Δ^ ^Δ^ ^CT^ was used (Livak and Schmittgen, 2001). Average CT for each sample was transformed into relative quantities, normalized with the respective factor, and standardized against CTRL diet.

**TABLE 3:** Oligonucleotide primers used in the reverse transcription-qPCR analysis and respective annealing temperature (°C), PCR efficiency (%), accession number and reference.

Gene	Primer sequence 5′-3′		Annealing T (°C)	PCR efficiency (%)	Accession number	References
β*actin*	F	TGCGTGACATCAAGGAGAAG	58	91	X89920.1	Avella et al., 2010
	R	CAGGACTCCATACCGAGGAA				
*ef1*α	F	CTGTCAAGGAAATCCGTCGT	60	100	AF184170.1	Estruch et al., 2018
	R	TGACCTGAGCGTTGAAGTTG				
*rps18*	F	AGGGTGTTGGCAGACGTTAC	60	101	AM490061.1	Salmerón et al., 2016
	R	CTTCTGCCTGTTGAGGAACC				
*Igf2*	F	TGGGATCGTAGAGGAGTGTTGT	62	92	AY996778.1	Benedito-Palos et al., 2007
	R	CTGTAGAGAGGTGGCCGACA				
*selk*	F	TGTACGTGTCCAACGGTCAG	60	95	XM_030423889.1	–
	R	TGAATTCCACTGCTCCCCAG				
*selp*	F	CATTTATGACAGGTGTGGCCG	62	94	XM_030416800.1	–
	R	CGCTTGCAGTACGCATCTTT				
*dio1*	F	GACTCGTCAGGGACTTCAGC	60	97	DQ888894.1	–
	R	GAGCCTCTCAGGCATAGCAC				
*dio2*	F	GGCAGTTGGTTGAGGACTTC	60	91	DQ888895.1	–
	R	TCCATACAGTCAGCCACCAG				
*gpx1a*	F	TGGAGAAGGTGGATGTGAATG	58	92	KC201352.1	Malandrakis et al., 2014
	R	GCCATCAGGACCAACAAGG				
*gpx1b*	F	GCGTTACAGCAGGAGGTT	60	90	KC201353.1	Malandrakis et al., 2014
	R	TGAGCACATCTGGTCATTCA				
*gpx4b*	F	ACGGCTTGTCTGTAAACTC	60	99	KC201354.1	Malandrakis et al., 2014
	R	GCTGGTGAATGTCCTCTC				
*cpt1a*	F	GTGCCTTCGTTCGTTCCATGATC	62	99	JQ308822.1	Betancor et al., 2016
	R	TGATGCTTATCTGCTGCCTGTTTG				
*Fas*	F	TGCCATTGCCATAGCACTCA	58	92	JQ277708.1	Houston et al., 2017
	R	ACCTTTGCCCTTTGTGTGGA				
*srebp1*	F	AGGGCTGACCACAACGTCTCCTCTCC	60	93	JQ277709.1	Houston et al., 2017
	R	GCTGTACGTGGGATGTGATGGTTTGGG				
*fads2*	F	GCAGGCGGAGAGCGACGGTCTGTTCC	60	99	AY055749.1	Betancor et al., 2016
	R	AGCAGGATGTGACCCAGGTGGAGGCAGAAG				
*elovl5*	F	CCTCCTGGTGCTCTACAAT	60	92	AY660879.1	Betancor et al., 2016
	R	GTGAGTGTCCTGGCAGTA				

*Reference genes: βactin, actin beta; ef1α, elongation factor 1 alpha and rps18, ribosomal protein S18. Target genes related to growth: igf2, insulin-like growth factor 2; selenoprotein genes: selk, selenoprotein K and selp, selenoprotein P; genes involved in iodine metabolism: dio1, iodothyronine deiodinase 1 and dio2, iodothyronine deiodinase 2; genes involved in oxidative stress: gpx1a, glutathione peroxidase 1a; gpx1b, glutathione peroxidase 1b and gpx4b, glutathione peroxidase 4b; and genes associated with lipid metabolism: cpt1a, carnitine palmitoyl transferase 1a; fas, fatty acid synthase; srebp1, sterol regulatory element binding protein 1; fads2, fatty acid desaturase 2; and elovl5, elongation of very long chain fatty acids protein 5.*

### Liver Preparation and Oxidative Stress Enzymatic Activity

Liver samples (*n* = 9 per treatment) were homogenized in phosphate-buffered saline (PBS, pH 7.4) using a tissue to PBS ratio of 1:15 (IKA T25 digital; Ultra Turrax). The homogenate was centrifuged at 13,500 × *g* for 15 min at 4°C (Z 383 K Hermle) and the resulting supernatant was aliquoted and stored at –80°C for determination of oxidative stress enzymatic activity. Analyses were performed in triplicate.

Tissue protein contents were determined according to the Bradford method using bovine serum albumin (BSA) as the standard (Bradford, 1976), and absorbances were measured at 595 nm. Superoxide dismutase (SOD) activity (EC 1.15.1.1) was carried out as described by Sun et al. (1988), using nitroblue tetrazolium (NBT) and xanthine oxidase (XOD), with 10 μL of liver extract and 250 μL of substrate (200 μL xanthine + 50 μL XOD), and a reaction time of 10 min. Samples absorbances were read at 550 nm and results are presented as units of SOD mg protein^–1^, where 1 unit of enzyme equals 50% NBT inhibition (% inhibition = Δ control – Δ sample)/Δ control × 100; Δ = abs min^–1^). Catalase (CAT) activity (EC 1.11.1.6) was determined based on consumption of hydrogen peroxide (H_2_O_2_, substrate; 250 μL), observed as the linear decrease in absorbance (240 nm) over time, according to the method of Beers and Sizer (1952), adapted for microplates by Li and Schellhorn (2007). A sample volume of 10 μL was used, and H_2_O_2_ consumption was monitored every 30 s for 5 min at 240 nm and 25°C. Results are expressed in μmol min^–1^ mg^–1^ protein. Selenium-independent and dependent glutathione peroxidase (GPx and SeGPx) activities (EC 1.11.1.9) were evaluated spectrophotometrically using a coupled assay based on glutathione reductase (Flohe and Gunzler, 1984). Hydrogen peroxide and cumene hydroperoxide substrates (200 μL buffer + 50 μL substrate), with 10 μL liver extract, were used to analyze activities of SeGPx and GPx, respectively, following the decrease in NADPH consumed during re-formation of GSH from GSSG at 340 nm, for 5 min at 25°C. SeGPx activity was calculated as μmol oxidized NADPH min^–1^ mg^–1^ protein and GPx as nmol NADPH oxidized min^–1^ mg^–1^ protein, using a molar extinction coefficient of 6.22 mM^–1^ cm^–1^. Lipid peroxidation (LPO) concentrations were assessed by measuring malondialdehyde (MDA) using the method described by Erdelmeier et al. (1998), which is based on the reaction of a chromogenic compound, 1-methyl-2-phenylindole (C_15_H_13_N). Two moles of this reagent react with one mole of MDA at 45°C for 60 min to form a stable chromophore with a maximum absorbance at 586 nm.

### Calculations

Growth performance calculations: Weight gain = final body weight (FBW) – initial body weight (IBW); Average body weight (ABW) = (FBW + IBW)/2; Condition factor (K) = 100 × (FBW/final body length^^3^); Specific growth rate (SGR) = (ln FBW – ln IBW)/days of the experiment × 100; Feed conversion ratio (FCR) = dry feed intake/(FBW – IBW); Protein efficiency ratio (PER) = (FBW – IBW)/crude protein intake; Hepatosomatic index (HSI) = 100 × (liver weight/FBW); Viscerosomatic index (VSI) = 100 × (weight of viscera/FBW).

Nutrient intake, retention, and gain calculations: Protein (P), lipids (L), or energy (E) intake = DM intake × P, L, E (kJ g^–1^) in the diet; DM retention/consumption = (FBW × final whole-body DM content – IBW × initial whole-body DM content)/dry feed consumption per fish; P, L, E or fatty acids (FAs) retention/consumption = 100 × (FBW × final whole-body P, L, E or FAs content in wet weight - IBW × initial whole-body P, L, E or FAs content in wet weight)/dry feed consumption per fish × P, L, FAs or E (kJ g^–1^) in the diet in% DM; DM, P, L, E or FAs gain = (FBW × final whole-body DM, P, L, E or FAs content in wet weight x%) – (IBW × initial whole-body DM, P, L, E or FAs content in wet weight x%)/ABW (kg)/days of the experiment.

### Statistical Analysis

Statistical analyses were performed using IBM SPSS^®^ Statistics 25.0 software (SPSS Inc., Chicago, IL, United States). All variables were tested for normality and homogeneity of variances using Kolmogorov-Smirnov and Levene’s tests, respectively, followed by one-way analysis of variance (ANOVA). A Tukey’s multiple comparison test was applied to compare individual means. Data were log-transformed when normality and/or homogeneity of variances assumptions were not met and a non-parametric test (Kruskal–Wallis *H*-test) was used when required. Significant differences were considered for a *P* < 0.05. A Spearmen’s rank correlation coefficient (*r*s) test was performed and significant correlations between different variables were considered at 0.05 (^∗^) or 0.01 (^∗∗^).

## Results

### Growth Performance and Nutrient Utilization

The growth performance parameters are displayed in Table 4. All biofortified diets were well accepted by the gilthead seabream. At the end of 12 weeks of feeding, fish fed the CTRL diet reached a FBW of 625.8 ± 5.4 g, that was similar to that of fish fed with AD1 and AD2 (622.6 ± 6.8 g and 623.1 ± 3.2 g, respectively). But fish fed diet AD3 had a significantly lower FBW (589.5 ± 5.5 g) than fish consuming any other diet. Final condition factor (K), feed conversion ratio (FCR), and specific growth rate (SGR) remained unaffected by diet. Nonetheless, a tendency for increased FCR in fish fed diet AD3 was observed (*p* = 0.06). Likewise, hepatosomatic (HSI) and viscerosomatic (VSI) indices were not significantly different among treatments. Protein and energy intake did not differ between diets, but lipid intake was significantly lower in fish fed AD3 compared with the CTRL diet. Weight gain (WG) and protein efficiency ratio (PER) of fish fed AD3 were also significantly lower compared with those fed the CTRL diet. Iodine and selenium intake were significantly higher in fish fed the AD diets, with the highest values being observed in fish fed AD3.

**TABLE 4:** Growth performance and somatic indexes of gilthead seabream fed the experimental diets for 85 days.

	CTRL	AD1	AD2	AD3	*P*
**Growth**					
Initial body weight (g, IBW)	371.20 ± 14.50	378.95 ± 2.38	375.52 ± 13.39	369.97 ± 9.82	0.75
Final body weight (g, FBW)	625.76 ± 5.38^a^	622.61 ± 6.77^a^	623.11 ± 3.19^a^	589.46 ± 5.49^b^	<0.001
Weight gain (g, WG)	254.56 ± 11.18^a^	243.66 ± 8.25^ab^	247.59 ± 16.33^ab^	219.49 ± 12.26^b^	0.04
Final condition factor (K)	1.84 ± 0.05	1.89 ± 0.13	1.79 ± 0.03	1.78 ± 0.02	0.30
Specific growth rate (SGR)	0.73 ± 0.05	0.69 ± 0.02	0.70 ± 0.06	0.65 ± 0.04	0.23
Feed conversion ratio (FCR)	1.67 ± 0.02	1.72 ± 0.06	1.73 ± 0.12	1.91 ± 0.13	0.06
Protein efficiency ratio (PER)	1.20 ± 0.03^a^	1.15 ± 0.04^ab^	1.14 ± 0.07^ab^	1.03 ± 0.07^b^	0.03
**Intake (g or kJ or mg kg^–1^ ABW day^–1^)**					
Dry matter (g kg^–1^)	9.90 ± 0.39	9.85 ± 0.05	10.13 ± 0.19	10.40 ± 0.23	0.09
Protein (g kg^–1^)	5.00 ± 0.19	4.97 ± 0.03	5.14 ± 0.10	5.24 ± 0.12	0.10
Lipids (g kg^–1^)	1.65 ± 0.06^a^	1.60 ± 0.01^ab^	1.59 ± 0.04^ab^	1.54 ± 0.03^b^	0.04
Energy (kJ kg^–1^)	236.02 ± 9.19	235.89 ± 1.31	242.50 ± 4.61	247.23 ± 5.43	0.12
Iodine (mg kg^–1^)	13.31 ± 0.52^d^	79.76 ± 0.44^c^	90.88 ± 1.73^b^	151.78 ± 3.34^a^	<0.001
Selenium (mg kg^–1^)	7.50 ± 0.29^c^	11.36 ± 0.06^b^	10.82 ± 0.21^b^	14.63 ± 0.32^a^	<0.001
**Somatic indexes**					
Hepatosomatic index (HSI, %)	1.56 ± 0.12	1.31 ± 0.07	1.29 ± 0.05	1.34 ± 0.17	0.07
Viscerosomatic index (VSI, %)	5.70 ± 0.44	5.60 ± 0.32	5.75 ± 0.9	5.73 ± 0.09	0.97

*Values are presented as mean ± standard deviation (SD). Growth performance and intake parameters were calculated with three pooled samples per treatment of six fish per tank. Somatic indexes were calculated with 15 fish per treatment (five fish per tank). Different superscript letters in a row denote significant differences among treatments.*

Iodine intake was negatively correlated with FBW and PER, while selenium intake presented a negative correlation with FBW, WG and PER, and a positive correlation with FCR (Supplementary Table 2).

### Whole-Body Composition, Fatty Acid Profile and Utilization

**FIGURE 1:**
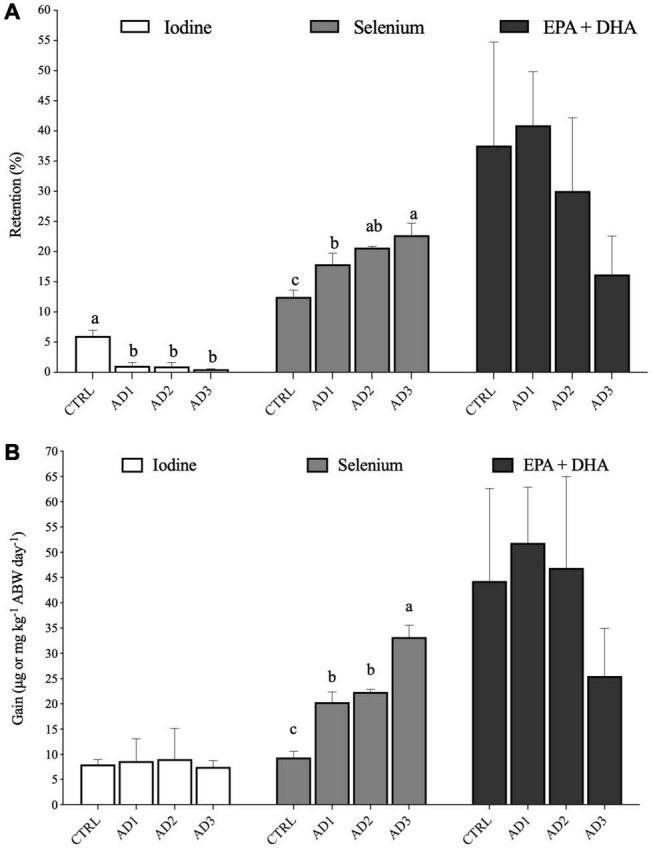
**(A)** Retention (% feed intake) and **(B)** Gain of iodine and selenium (μg kg^−1^ ABW day^−1^) and of EPA + DHA (mg kg^−1^ ABW day^−1^). EPA + DHA = eicosapentaenoic acid + docosahexaenoic acid. Values are presented as mean ± SD. Different letters denote significant differences among treatments.

Whole-body composition, retention and gain in protein, lipids, energy were not significantly affected by diet (Table 5). Retention and gain in selenium, as well as whole-body composition (Table 5 and Figure 1), were significantly higher in fish consuming the AD diets, with fish fed the AD3 diet presenting the highest values. For iodine, although retention was significantly decreased in fish fed the AD diets compared to CTRL, no significant differences could be observed on whole-body composition and gain among dietary treatments.

**TABLE 5:** Whole-body composition and nutrient retention and gain of gilthead seabream fed the experimental diets for 85 days.

	CTRL	AD1	AD2	AD3	*P*
**Whole-body composition**					
Moisture (% WW)	59.61 ± 0.66	60.14 ± 0.12	60.63 ± 0.95	59.87 ± 0.96	0.44
Ash (% WW)	3.79 ± 0.24	3.74 ± 0.18	3.76 ± 0.08	3.72 ± 0.15	0.97
Protein (% WW)	18.07 ± 0.46	18.35 ± 0.03	18.61 ± 0.30	18.18 ± 0.40	0.30
Lipids (% WW)	15.27 ± 0.43	15.69 ± 1.28	14.67 ± 1.23	14.52 ± 0.84	0.49
Energy (kJ g^–1^ WW)	10.94 ± 0.32	11.11 ± 0.43	10.7 ± 0.35	11.02 ± 0.37	0.60
Iodine (mg kg^–1^ WW)	0.19 ± 0.01	0.20 ± 0.03	0.20 ± 0.04	0.20 ± 0.01	0.96
Selenium (mg kg^–1^ WW)	0.24 ± 0.001^c^	0.32 ± 0.01^b^	0.33 ± 0.01^b^	0.42 ± 0.02^a^	<0.001
**Retention (% of Intake)**					
Dry matter	28.40 ± 1.40	26.68 ± 0.62	25.63 ± 0.50	24.43 ± 2.73	0.08
Protein	21.96 ± 1.24	21.85 ± 0.65	22.29 ± 2.05	19.12 ± 1.49	0.09
Lipids	68.81 ± 4.60	72.49 ± 10.47	64.25 ± 9.87	61.31 ± 10.90	0.51
Energy	33.52 ± 2.44	33.34 ± 1.72	30.45 ± 1.46	29.92 ± 3.75	0.24
**Gain (g or kJ kg^–1^ ABW day^–1^)**					
Dry matter	2.82 ± 0.24	2.63 ± 0.05	2.60 ± 0.10	2.54 ± 0.26	0.35
Protein	1.10 ± 0.04	1.09 ± 0.03	1.15 ± 0.12	1.00 ± 0.06	0.16
Lipids	1.14 ± 0.11	1.16 ± 0.17	1.02 ± 0.14	0.94 ± 0.15	0.30
Energy	0.79 ± 0.08	0.79 ± 0.05	0.74 ± 0.05	0.74 ± 0.09	0.66

*Values are presented as mean ± SD, with three pooled samples per treatment of six fish per tank. Different superscript letters in a row denote significant differences among treatments.*

*ABW, average body weight; WW, wet weight.*

*Whole-body initial composition: 64.02 moisture (% WW); 35.98 DM (%); 4.32 ash (% WW); 17.94 protein (% WW); 12.78 lipids (% WW); 9.41 energy (kJ g^–1^).*

The whole-body fatty acid profile (% total fatty acids; Table 6) indicated that the most abundant fatty acids were OA (18:1 n-9 c, 34–35%) followed by palmitic acid (16:0, 21-26%) and LA (18:2 n-6 c, 13-17%), independently of the diet. Fish fed AD1 had significantly lower concentrations of myristic acid (14:0) compared to those fed AD3. Fish fed AD1 also had the highest PUFAs content, which was significantly higher than those fed AD3. Such differences were largely due to significantly higher concentrations of α-linolenic acid (ALA, 18:3 n-3) and EPA in the whole-body of fish fed AD1 in comparison with AD3. The whole-body DHA increased significantly in fish fed AD1 and AD2 compared to either CTRL or AD3 diets, reaching the highest values in fish fed AD1 diet.

**TABLE 6:** Whole-body fatty acid composition (% total fatty acids) and retention (% feed intake).

	CTRL	AD1	AD2	AD3	*P*
**Fatty acids**					
14:0	3.08 ± 0.33^ab^	2.71 ± 0.31^b^	3.27 ± 0.17^ab^	3.59 ± 0.20^a^	0.02
16:0	22.57 ± 2.45	20.57 ± 2.57	23.38 ± 1.29	26.13 ± 2.06	0.07
18:0	5.54 ± 0.96	4.89 ± 0.65	5.50 ± 0.18	6.33 ± 0.76	0.17
^1^Σ SFA	32.36 ± 3.72	29.41 ± 3.58	33.46 ± 1.80	37.30 ± 2.91	0.07
16:1 n-7	4.04 ± 0.72	4.45 ± 0.45	4.36 ± 0.25	4.26 ± 0.61	0.81
18:1 n-9 c (OA)	35.42 ± 0.68	35.16 ± 0.81	34.17 ± 1.51	34.22 ± 0.51	0.33
20:1 n-9	1.60 ± 0.06	1.34 ± 0.16	1.53 ± 0.03	1.55 ± 0.06	0.11
^2^Σ MUFA	41.95 ± 1.04	41.91 ± 1.10	40.91 ± 1.63	40.84 ± 0.97	0.55
18:2 n-6 c (LA)	16.06 ± 2.05	16.79 ± 1.49	15.38 ± 0.30	13.43 ± 1.62	0.11
18:3 n-3 (ALA)	3.45 ± 0.52^ab^	3.83 ± 0.43^a^	3.24 ± 0.15^ab^	2.68 ± 0.45^b^	0.04
20:5 n-3 (EPA)	1.74 ± 0.13^ab^	1.78 ± 0.17^a^	1.74 ± 0.07^ab^	1.36 ± 0.20^b^	0.03
22:6 n-3 (DHA)	2.92 ± 0.28^c^	4.36 ± 0.38^a^	3.70 ± 0.14^b^	2.85 ± 0.04^c^	<0.001
^a^EPA + DHA	4.66 ± 0.33^bc^	6.14 ± 0.54^a^	5.44 ± 0.15^ab^	4.21 ± 0.16^c^	<0.001
^3^Σ PUFA	25.40 ± 2.66^ab^	28.08 ± 2.52^a^	25.22 ± 0.32^ab^	21.46 ± 2.25^b^	0.03
**Retention (% of Intake)**					
14:0	83.50 ± 14.23	48.73 ± 12.11	57.42 ± 14.73	62.89 ± 19.40	0.11
16:0	117.57 ± 27.58^a^	52.11 ± 16.09^b^	64.48 ± 17.31^ab^	74.37 ± 27.02^ab^	0.04
18:0	115.21 ± 35.27	68.88 ± 25.12	86.55 ± 33.97	105.96 ± 47.77	0.46
^1^Σ SFA	108.46 ± 24.88	52.84 ± 15.81	64.73 ± 18.17	74.22 ± 27.11	0.07
16:1 n-7	49.22 ± 39.50	40.99 ± 17.64	41.09 ± 38.66	32.04 ± 30.17	0.93
18:1 n-9 c (OA)	64.32 ± 25.10	47.18 ± 8.08	53.98 ± 31.26	49.46 ± 26.79	0.83
20:1 n-9	63.24 ± 16.75	29.95 ± 17.95	51.24 ± 24.26	50.51 ± 28.64	0.39
^2^Σ MUFA	57.52 ± 23.11	41.95 ± 7.44	47.06 ± 28.04	42.71 ± 24.11	0.81
18:2 n-6 c (LA)	44.95 ± 21.31	37.05 ± 10.12	35.39 ± 15.89	22.06 ± 11.75	0.39
18:3 n-3 (ALA)	42.64 ± 17.35	41.27 ± 9.09	35.91 ± 14.12	24.26 ± 7.38	0.34
20:5 n-3 (EPA)	30.47 ± 10.57	30.34 ± 5.87	25.22 ± 9.47	15.59 ± 4.38	0.16
22:6 n-3 (DHA)	47.62 ± 27.03	48.78 ± 11.15	33.94 ± 14.39	16.75 ± 8.83	0.15
^3^Σ PUFA	42.42 ± 18.81	37.89 ± 9.27	32.66 ± 13.69	20.29 ± 9.24	0.28

*Values presented as mean ± SD, with three pooled samples per treatment of six fish per tank for final whole-body composition and two pooled samples of six fish per tank for initial whole-body composition. Different superscript letters in a row denote significant differences among treatments.*

*^1^Σ SFA is the sum of saturated fatty acids and also includes 15:0, 17:0, 20:0, 22:0 and 24:0;*

*^2^Σ MUFA is the sum of monounsaturated fatty acids and also includes 17:1 n-7, 22:1 n-9 and 24:1 n-9;*

*^3^Σ PUFA is the sum of polyunsaturated fatty acids and also includes 18:3 n-6, 20:2 n-6, 20:3 n-6 and 20:4 n-6;*

*^a^EPA + DHA = eicosapentaenoic acid + docosahexaenoic acid. OA: oleic acid; LA: linoleic acid; ALA: α-linolenic acid.*

Overall, fatty acid retention (Table 6 and Figure 1A) and gain (Supplementary Table 1 and Figure 1B) in gilthead seabream were not significantly affected by diet, except for 16:0, which was less retained in fish fed AD1 compared with those fed the CTRL diet. Moreover, fish fed AD1 had the lowest retention and gain of SFAs and MUFAs, while fish fed diet AD3 had the lowest PUFAs retention and gain, including EPA and DHA (although without statistical significance; Figure 1). The highest DHA retention and gain was observed in fish fed AD1, but again without statistical significance (Table 6 and Supplementary Table 1).

### Muscle Fatty Acid Profile

Muscle total lipids were not significantly affected by diet, while muscle fatty acid profiles differed significantly amongst the diets (Table 7). SFAs, specifically myristic and palmitic acids (14:0 and 16:0), were significantly higher in fish fed AD2 compared with those fed CTRL. MUFAs were significantly reduced in fish fed AD2 and AD3 in comparison with CTRL, mainly due to significantly lower concentrations of OA (18:1 n-9) in those fish. Fish fed AD2 also had the lowest muscle concentrations of LA (18:2n-6 c), which was significantly lower compared with CTRL. Muscle from fish fed AD2 and AD3 had significantly higher DHA concentrations compared with CTRL, with the sum of EPA and DHA being highest in fish fed AD3 (% total fatty acids). Fish fed AD2 and AD3 diets had the highest net content of EPA + DHA (0.7 g 100 g^–1^ of filet), being significantly higher than in fish fed the CTRL (0.5 g 100 g^–1^ of filet).

**TABLE 7:** Muscle total lipid content and fatty acid composition.

	CTRL	AD1	AD2	AD3	*P*
Total lipids (g 100 g^−1^ WW)	8.35 ± 0.34	8.27 ± 0.14	8.69 ± 0.76	8.97 ± 0.39	0.22
**Fatty acids (% total fatty acids)**					
14:0	2.47 ± 0.06^b^	2.56 ± 0.09^ab^	2.78 ± 0.17^a^	2.69 ± 0.09^ab^	0.04
16:0	18.15 ± 0.38^b^	19.90 ± 1.23^ab^	20.82 ± 0.84^a^	20.14 ± 0.68^ab^	0.02
18:0	4.48 ± 0.18	4.53 ± 0.24	4.64 ± 0.32	4.41 ± 0.23	0.74
^1^Σ SFA	26.09 ± 0.48^b^	28.01 ± 1.36^ab^	29.37 ± 1.20^a^	28.32 ± 0.84^ab^	0.03
16:1 n-7	4.42 ± 0.08	4.28 ± 0.07	4.44 ± 0.19	4.39 ± 0.20	0.56
18:1 n-9 c (OA)	35.93 ± 0.35^a^	35.29 ± 0.91^ab^	34.11 ± 0.34^b^	33.91 ± 0.41^b^	0.006
20:1 n-9	1.42 ± 0.02	1.44 ± 0.01	1.43 ± 0.04	1.43 ± 0.08	0.92
^2^Σ MUFA	42.82 ± 0.36^a^	42.02 ± 1.04^ab^	41.02 ± 0.31^b^	40.73 ± 0.45^b^	0.01
18:2 n-6 c (LA)	18.42 ± 0.39^a^	17.56 ± 0.81^ab^	16.64 ± 0.53^b^	17.53 ± 0.39^ab^	0.03
18:3 n-3 (ALA)	4.35 ± 0.37	3.87 ± 0.25	3.63 ± 0.22	3.95 ± 0.34	0.10
20:5 n-3 (EPA)	2.63 ± 0.18	2.18 ± 0.22	2.33 ± 0.16	2.46 ± 0.26	0.12
22:6 n-3 (DHA)	4.31 ± 0.24^b^	5.03 ± 0.44^ab^	5.60 ± 0.39^a^	5.64 ± 0.34^a^	0.006
^a^EPA + DHA	6.94 ± 0.12^b^	7.21 ± 0.23^ab^	7.92 ± 0.52^ab^	8.10 ± 0.60^a^	0.03
^3^Σ PUFA	30.90 ± 0.69	29.82 ± 1.17	29.40 ± 1.19	30.75 ± 1.04	0.30
**Fatty acids (g 100 g^–1^ WW)**					
EPA + DHA	0.53 ± 0.02^b^	0.55 ± 0.02^ab^	0.66 ± 0.08^a^	0.67 ± 0.02^a^	0.04

*Values presented as mean ± SD, with three pooled muscle samples per treatment of six fish per tank. Different superscript letters in a row denote significant differences among treatments.*

*^1^Σ SFA is the sum of saturated fatty acids and also includes 15:0, 17:0, 20:0, 22:0 and 24:0;*

*^2^Σ MUFA is the sum of mono-unsaturated fatty acids and also includes 17:1 n-7, 22:1 n-9 and 24:1 n-9;*

*^3^Σ PUFA is the sum of polyunsaturated fatty acids and also includes 18:3 n-6, 20:2 n-6, 20:3 n-6 and 20:4 n-6;*

*^a^EPA + DHA = eicosapentaenoic acid + docosahexaenoic acid.*

*OA, oleic acid; LA, linoleic acid; ALA, α-linolenic acid; WW, wet weight.*

### Gene Expression in Liver

**FIGURE 2:**
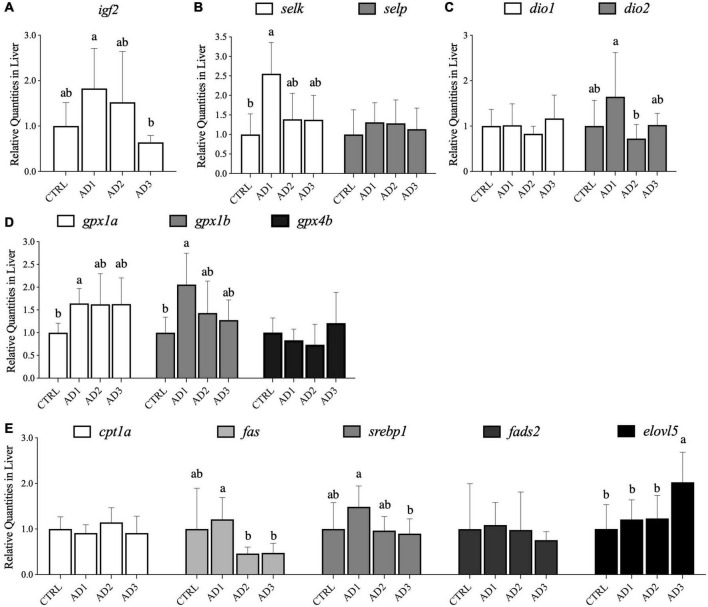
Gene expression in the liver of gilthead seabream fed the experimental diets over 12 weeks. **(A)** Genes growth-related; **(B)** genes involved in selenium metabolism; **(C)** genes involved in iodine metabolism; **(D)** genes associated with oxidative stress; **(E)** genes involved in lipid metabolism (lipolytic, lipogenic, desaturation, and elongation pathway related gene). Relative abundance in liver is presented as mean ± SD (*n* = 9 fish per treatment). Different letters denote significant differences among treatments.

Expression of a panel of genes related to growth, selenoproteins involved in selenium and iodine metabolism and oxidative stress, and lipid metabolism were assessed in liver from gilthead seabream (Figure 2). Concerning genes associated with growth, the *igf2* expression (Figure 2A) was significantly downregulated in the liver of fish fed AD3 compared with those fed AD1. Moreover, hepatic expression of *igf2* was significantly and positively correlated with protein retention and gain (Supplementary Table 2).

In liver, the expression of several selenoprotein genes was affected by diet (Figure 2B). Transcript levels of *selk* were significantly upregulated in fish fed AD1 compared with fish fed the CTRL diet, but transcription of *selp* remained unaffected by diet. Expressions of liver *selk* and *dio2* were also significantly and positively correlated with hepatic *igf2* expression (Supplementary Table 2). AD1 was associated with increased hepatic expression of a selenoprotein gene related to iodine metabolism (*dio2*; Figure 2C) compared to AD2. The oxidative stress genes *gpx1a* and *gpx1b* were significantly upregulated in fish fed AD1 compared with those fed CTRL (Figure 2D).

For genes involved in hepatic lipid metabolism, increased expression of lipogenesis pathway related genes (*fas* and *srebp1*) was observed in fish consuming the AD1 diet compared with the other AD diets, but not in those fed the CTRL. The gene associated with the lipolytic process, *cpt1a*, was not affected by the dietary treatments. Concerning desaturation and elongation pathway related genes, no differences were observed on *fads2* expression while *elovl5* was up regulated in fish that consumed AD3 in comparison with the other diets (Figure 2E). A significant and negative correlation was observed between *elovl5* expression and several growth performance parameters (FBW, WG, DGI, SGR, and PER), lipid intake, and protein retention and gain (Supplementary Table 2).

### Oxidative Stress Parameters in Liver

**FIGURE 3:**
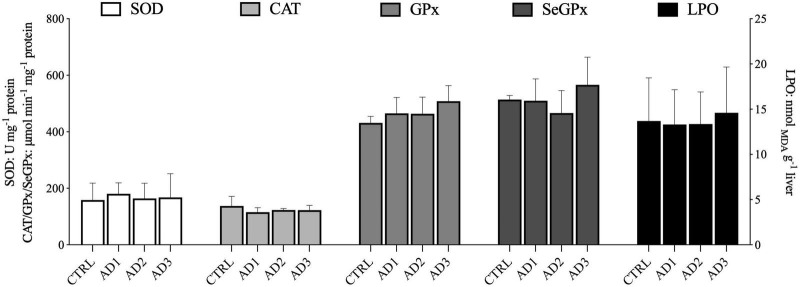
Oxidative stress-related parameters in the liver of gilthead seabream fed the experimental diets over 12 weeks. No significant differences between dietary treatments were observed. Values are presented as mean ± SD.

The oxidative stress parameters in liver are displayed in Figure 3. No significant differences in the activity of SOD, CAT, GPx, and SeGPx were observed between dietary treatments. Likewise, the LPO values were similar among fish fed the different experimental diets.

## Discussion

In recent years, efforts have been made to find natural, highly nutritious alternatives to replace FM and FO, which could simultaneously promote fish health and improve the nutritional quality of filets for human consumption. In the present study, fish fed diet AD1, with 10% FM and 4.4% FO, and with 20% less vegetable oils than the CTRL diet, grew equally well, indicating that a microalgae blend can be successfully used to partially replace FM and FO in diets for *S. aurata*. Moreover, a concomitant supplementation of seabream diets with 0.4% iodine-rich *L. digitata* and 0.02% selenised yeast can be achieved without negatively affecting fish growth. However, higher levels of *L. digitata* and selenised yeast resulted in lower seabream growth. The negative correlation observed between selenium and iodine intake and FBW and PER might be due to a decline in overall nutrient/energy bioavailability, which may have compromised nutrient absorption leading to growth impairment. Although digestibility of diets was not presently tested, previous studies with *S. latissima* associated growth impairment in rainbow trout with reduced nutrient digestibility and a reduction in tunica muscularis thickness, which could decrease intestine strength and motility (Granby et al., 2020). Very few studies evaluated the ability of fish to digest kelp, but according to Pereira et al. (2012), *Sargassum muticum* had comparatively lower nutrient and energy digestibility values compared to *Porphyra dioica*, *Ulva* spp., *Gracilaria vermiculophylla* in rainbow trout. The present results with gilthead seabream, although still preliminary, are encouraging, demonstrating the feasibility of using biofortified aquafeeds with algae and selenised yeast. But future studies should delve into the digestibility of these diets to have a more comprehensive perspective on the cost-benefit ratio of such feeds.

Somatotropic axis genes are reliable growth markers, and evidence suggests that both environmental and nutritional factors modulate expression and circulating concentrations of growth hormone receptors and insulin-like growth factors (*igfs*) (Pérez-Sánchez et al., 2018). Downregulation of hepatic *igf2* was observed in fish fed AD3, resulting in a significantly lower final body weight of these fish compared with CTRL. In a previous study evaluating the impact of FO replacement with VO in gilthead seabream, a significantly lower final body weight was observed in fish consuming the 100% VO diet compared to other diets (100% FO; 33% VO; and 66% VO), which was accompanied by a downregulation of hepatic *ig1* and *igf2* (Benedito-Palos et al., 2007). In the present study, *igf2* hepatic expression was positively correlated with protein retention and gain, which are major determinants of growth (Mommsen, 2001). Therefore, the downregulation of growth-related genes could have contributed to the growth impairment of fish fed AD3. But it would be necessary to measure the circulating concentrations of growth hormones and Igf1 to have a better understanding of nutritional modulation of the somatotropic axis.

The replacement of FO with VO in aquafeeds is known to reduce long-chain polyunsaturated fatty acids (LC-PUFAs), particularly eicosapentaenoic acid (EPA, 20:5n-3) and docosahexaenoic acid (DHA, 22:6n-3) (Nasopoulou and Zabetakis, 2012). This may negatively impact fish performance due to the reported limited capacity of a large variety of teleost fish lineages to synthesize LC-PUFAs (Tocher, 2015; Lopes-Marques et al., 2018). Yet, the replacement of 33% FM with microalgae meal and the inclusion of DHA-rich *Schizochytrium* sp. resulted in higher concentrations of DHA in biofortified diets, even in the case of AD1, which had an additional 20% reduction in FO. Moreover, none of the fortified diets significantly affected gilthead seabream whole-body composition, or nutrient retention and gain. Nevertheless, a trend for lower protein and lipid retention and gain in fish consuming AD3 may have contributed to their reduced growth.

In terms of essential fatty acids, the concomitant partial replacement of FM and FO either did not affect or have even increased whole-body and muscle EPA and DHA levels in fish. In fact, filets of fish consuming AD2 and AD3 diets presented the highest EPA + DHA contents, providing 2.6–2.7 times the recommended by EFSA 0.25 g per 100 g portion (EFSA, 2010). In general, muscle fatty acid profiles mirrored that of the diets, but whole-body trends were not the same. These results are indicative of selective retention of omega-3 fatty acids in muscle, which has been reported previously for farmed fish species (Mourente and Bell, 2006; Dantagnan et al., 2009). Together, our results indicate that DHA-rich *Schizochytrium* sp. is an effective substitute for FO in gilthead seabream diets since n-3 PUFAs were effectively retained in the muscle. In Nile tilapia (*Oreochromis niloticus*) and Atlantic salmon (*Salmo salar*), replacement of FO with *Schizochytrium* sp. meal resulted in increased DHA concentrations in filets (Miller et al., 2007; Sarker et al., 2016; Kousoulaki et al., 2020), further supporting the potential of LC-PUFA-rich microalgae as suitable candidates to replace FO in diets for farmed species. However, despite the slightly higher capacity of fish fed AD1 to retain DHA (49% *vs.* 48% in the CTRL) and EPA + DHA (41% *vs.* 38% in the CTRL) at whole-body level, differences were not significant among treatments. Ribeiro (2019) reported low retention values for EPA + DHA (17–24%) in gilthead seabream filets fed diets with 15–30% FM and 12–15% FO, with DHA (24–37%) being retained better than EPA (9–15%). In the present study, and although the concomitant replacement of FM and FO (AD1 diet) led to the highest retention values of EPA + DHA (non-significant), new strategies must be developed to ensure higher retention of essential LC-PUFAs, since these values are still very low.

Previous studies have indicated that, like many marine fish, gilthead seabream cannot synthesize EPA and DHA efficiently (Tocher, 2015; Castro et al., 2016). Nevertheless, nutritional modulation of genes encoding LC-PUFAs biosynthetic enzymes has also been reported (Izquierdo et al., 2008). A previous study in black seabream (*Acanthopagrus schlegelii*) showed decreased expression of elongation of very long-chain fatty acids protein 5 (*elovl5*) in response to increased dietary n-3 LC-PUFAs (Jin et al., 2017a) or high DHA:EPA ratios (Jin et al., 2017b). Compared with CTRL, AD3 diet had higher concentrations of n-3 LC-PUFAs and a higher DHA:EPA ratio (1.2 for AD3 vs. 0.7 for CTRL). Therefore, the observed upregulation of hepatic *elovl5* in the present study might be a metabolic response to the lower bioavailability of LC-PUFAs in fish consuming AD3. The LC-PUFAs biosynthetic pathway is highly dependent on sequential steps of desaturation and elongation of C18 PUFAs (Tocher, 2010) and the increased hepatic expression of *elovl5* was accompanied by decreased whole-body C18 PUFAs and increased muscle n-3 LC-PUFAs concentrations. This suggests that fish fed AD3 elongated more C18 PUFAs to produce n-3 LC-PUFAs that were further retained by the muscle. Overall, our results support the hypothesis of a nutritional regulation of genes involved in the synthesis of LC-PUFAs. Additionally, a balance between the lipogenic and lipolytic processes is essential for lipid metabolism homeostasis. In the present study, the lipolytic pathway related gene (*cpt1a*) remained unaffected by the dietary treatments. In contrast, an upregulation of the lipogenic genes, *fas* and *srebp1*, was observed in response to the AD1 diet (with 20% reduction of FO), but only when compared with the other AD diets, not differing from the CTRL. Therefore, the concomitant replacement of FM and FO by microalgae is achievable without major impacts on both anabolic and catabolic processes associated with lipid metabolism.

Selenium and iodine are essential nutrients for human consumption and strategies to fortify fish in those elements are of particular relevance. In the present study, fish fed the diets supplemented with selenised yeast were able to increasingly retain the ingested selenium and accumulate it in their bodies. In a complementary study, with the same enriched feeds, it was demonstrated that the dietary supplementation with 0.02% selenised yeast was enough to achieve higher selenium contents in seabream filets compared with CTRL (0.23–0.36 μg g^–1^ in AD diets vs. 0.18 μg g^–1^ in CTRL), without increasing consumer’s exposure to toxic elements (Barbosa et al., 2020). Together, these results indicate that organic selenium provided by selenised yeast is an effective vehicle to fortify gilthead seabream filets. Contrarily to selenium, iodine gain remained similar among diets, but its retention efficiency was comprised by the higher levels of iodine in diets supplemented with *L. digitata.* In fact, it was shown that only the highest incorporation of *L. digitata* (0.8%) in AD3 fortified the filet with iodine (0.09 μg g^–1^ in AD3 vs. 0.07 μg g^–1^ in CTRL) (Barbosa et al., 2020), but impaired fish growth. Therefore, further studies are required to find sustainable feed formulations that can efficiently fortify the filets of gilthead seabream in iodine without compromising growth performance.

Dietary selenium is involved in several fish physiological processes, namely growth and antioxidant defense mechanism, through the action of selenoproteins such as deiodinases and glutathione peroxidase (Khan et al., 2017). In the present study, the hepatic expression of several selenoproteins (*selk*, *dio2*, *gpx1a*, and *gpx1b*) was evaluated and, generally, an upregulation was observed in fish consuming AD1, so the response of selenoproteins to dietary selenium level was not dose-dependent. Deiodinase enzymes have an important role in iodine and thyroid hormones metabolism, converting the inactive thyroxine (T4) to the active form triiodothyronine (T3) (Milanesi and Brent, 2017). The expression of the hepatic *dio2* followed the same trend observed for most selenoproteins rather than reflecting different iodine concentrations of diets supplemented with *L. digitata*. These results are in agreement with a previous study with rainbow trout, where diets containing high concentrations of iodine, through supplementation with the macroalga *S. latissima*, had limited impact on iodine-metabolism-related genes (Ferreira et al., 2020). In the present study, the expression of *dio2* was correlated positively with the expression of hepatic *igf2*, indicating that the effect of dietary selenium on growth might be partly exerted through the action of deidodinases. The determination of circulating concentrations of T3 and T4 could provide further insights into the impact of diets supplemented with organic selenium and iodine on thyroid hormone metabolism in fish.

As rich sources of bioactive compounds, the inclusion of algae in aquafeeds is often associated with protective effects against oxidative damage (Goiris et al., 2012; Wan et al., 2019). However, data from this study indicate a lack of differences in oxidative stress enzymes and LPO in the liver, which suggests that the experimental diets had limited impact on the antioxidant status, and that fish were able to maintain homeostasis. Antioxidant enzymes such as CAT, GPx and SeGPx are paramount to lessen the negative effects of hydroperoxides, increasing the body’s ability to protect cells from oxidative damage, thus preventing lipid peroxidation. The expression of both *gpx1a* and *gpx1b*, which encode for glutathione peroxidase, increased in the liver of fish fed AD1. However, the differences at the phenotype level (activity of oxidative stress enzymes) did not reflect the genotypic differences observed in the liver. Since all fish were reared under optimal environmental conditions for the species, a pro-oxidant challenge should have been carried out to further clarify potential health benefits associated with the tested diets, especially if complemented with a quantification of liver glutathione. Additionally, the simultaneous upregulation of several genes encoding selenoproteins (*gpx1*, *selk*, and *dio2*) in fish consuming the AD1 diet may indicate that a prolonged exposure to this diet or a pro-oxidant challenge may modulate the levels of these proteins.

## Conclusion

Our results demonstrated that biofortified diets with up to 0.4% *L. digitata* and 0.02% selenised yeast do not affect fish performance, but higher concentrations (0.8 and 0.04%, respectively) impair gilthead seabream growth. Fish consuming AD1 and AD2 grew equally well as fish fed the CTRL diet, indicating the potential of microalgae meal (*Chlorella* sp., *Tetraselmis* sp., and DHA-rich *Schizochytrium* sp.) to partially replace both FM and FO in diets for *S. aurata*. LC-PUFAs retention values were generally low, highlighting the need for new approaches to improve it in fish. Moreover, the high price and unpredictable supply of *Schizochytrium* sp. is still a limiting factor for global implementation in aquafeeds. Therefore, further efforts should be made to either reduce these costs or produce cost-effective feeds combining LC-PUFAs rich-microalgae supplementation with more affordable plant-based ingredients. A concomitant decreased bioavailability of LC-PUFAs and upregulation of hepatic *elovl5* in fish consuming AD3 resulted in higher EPA + DHA contents in the muscle. In fact, the filet of fish consuming this diet contained EPA and DHA levels that were 2.7 times above the minimum recommended levels for human consumption. Additionally, the experimental diets had limited effects on the lipogenic (*fas* and *srebp1*) and lipolytic (*cpt1a*) pathways related genes. Overall, omega-3 fatty acids from microalgae were effectively accumulated in *S. aurata* muscle, confirming their potential as a good source of dietary lipids for this fish species. A higher retention and gain of selenium were observed for fish consuming the AD diets, indicating the potential of selenised yeast to fortify fish in selenium. Supplementation with *L. digitata* led to similar iodine gain, but lower retention levels of iodine, evidencing the need for new approaches to fortify gilthead seabream in this essential element. Despite the absence of differences in antioxidant enzymes activities, hepatic expression of *gpx1a* and *gpx1b* was upregulated in fish fed AD1. Likewise, other selenoproteins genes (*selk*, *dio2*) were also upregulated in fish consuming this diet. Further studies seem to be required under challenging conditions to further evaluate the health benefits of the tested diets.

## Data Availability Statement

The raw data supporting the conclusions of this article will be made available by the authors, without undue reservation.

## Ethics Statement

The animal study was reviewed and approved by Ethical Committee of the EPPO-IPMA (Estação Piloto de Piscicultura de Olhão, Av. Parque Natural da Ria Formosa N, 8700-194, Olhão), overseen by the National Competence Authority.

## Author Contributions

MF: manuscript draft, investigation, formal analysis, and writing – review and editing. PR: investigation and formal analysis. LR: conceptualization, methodology, investigation, and writing – review and editing. MB: investigation. VD, SS, CS, and AlM: investigation and writing – review and editing. PP-F: funding acquisition and methodology. JD: conceptualization. LC: investigation, methodology, and writing – review and editing. AnM and MN: funding acquisition, methodology, and writing – review and editing. LV: conceptualization, methodology, validation, and writing – review and editing. All authors contributed to the article and approved the submitted version.

## Conflict of Interest

JD was employed by the company Sparos Lda. The remaining authors declare that the research was conducted in the absence of any commercial or financial relationships that could be construed as a potential conflict of interest.

## Publisher’s Note

All claims expressed in this article are solely those of the authors and do not necessarily represent those of their affiliated organizations, or those of the publisher, the editors and the reviewers. Any product that may be evaluated in this article, or claim that may be made by its manufacturer, is not guaranteed or endorsed by the publisher.

## Footnotes

1
www.qbaseplus.com
