# Supplementary material for: Biofortified Diets Containing Algae and Selenised Yeast: Effects on Growth Performance, Nutrient Utilization, and Tissue Composition of Gilthead Seabream (Sparus aurata)
Source: Front Physiol. 2022 Jan 13;12:812884. doi: 10.3389/fphys.2021.812884 (PMC8793800; doi:10.3389/fphys.2021.812884)
Supplement: Supplementary file 1 [file Data_Sheet_1.docx]

Supplementary Material

Supplementary Table 1. Fatty acid gain of gilthead seabream fed the experimental diets for 85 days.

| **Gain (mg kg^-1^ ABW day^-1^)** | **CTRL** | **AD1** | **AD2** | **AD3** | ***P*** |
| --- | --- | --- | --- | --- | --- |
| 14:0 | 33.98 ± 5.12 | 19.84 ± 4.90 | 30.90 ± 7.48 | 34.63 ± 10.04 | 0.11 |
| 16:0 | 226.91 ± 48.63 | 132.32 ± 40.63 | 194.18 ± 49.26 | 227.08 ± 78.76 | 0.21 |
| 18:0 | 53.33 ± 16.12 | 27.85 ± 10.11 | 42.03 ± 15.82 | 52.44 ± 22.91 | 0.29 |
| ^1^ ∑ SFA | 324.81 ± 68.73 | 188.47 ± 56.08 | 277.58 ± 73.90 | 323.22 ± 112.73 | 0.21 |
| 16:1 n-7 | 20.93 ± 16.32 | 14.33 ± 6.13 | 17.80 ± 16.48 | 13.00 ± 14.44 | 0.94 |
| 18:1 n-9 c | 294.05 ± 102.30 | 183.43 ± 30.47 | 204.85 ± 115.17 | 190.32 ± 99.60 | 0.48 |
| 20:1 n-9 | 13.27 ± 2.97 | 4.82 ± 2.88 | 9.04 ± 4.12 | 8.98 ± 4.94 | 0.14 |
| ^2^ ∑ MUFA | 334.81 ± 120.10 | 207.27 ± 35.69 | 235.51 ± 136.32 | 216.39 ± 118.09 | 0.50 |
| 18:2 n-6 c | 145.51 ± 63.32 | 107.27 ± 28.82 | 101.49 ± 43.81 | 64.50 ± 33.17 | 0.25 |
| 18:3 n-3 | 42.71 ± 15.69 | 37.98 ± 8.20 | 32.57 ± 12.26 | 22.68 ± 6.61 | 0.23 |
| 20:5 n-3 (EPA) | 20.94 ± 6.38 | 16.34 ± 3.09 | 17.48 ± 6.27 | 10.94 ± 2.91 | 0.18 |
| 22:6 n-3 (DHA) | 23.36 ± 12.32 | 35.51 ± 7.95 | 29.40 ± 11.94 | 14.58 ± 7.43 | 0.15 |
| ^3^ ∑ PUFA | 241.86 ± 97.29 | 204.35 ± 49.09 | 186.64 ± 75.01 | 117.95 ± 51.47 | 0.26 |

Values presented as mean ± SD, with 3 pooled samples per treatment of 6 fish per tank. No significant differences between dietary treatments were observed.

^1^ ∑ SFA is the sum of saturated fatty acids and also includes 15:0, 17:0, 20:0, 22:0 and 24:0; ^2^ ∑ MUFA is the sum of mono-unsaturated fatty acids and also includes 17:1 n-7, 22:1 n-9 and 24:1 n-9; ^3^ ∑ PUFA is the sum of polyunsaturated fatty acids and also includes 18:3 n-6, 20:2 n-6, 20:3 n-6 and 20:4 n-6; EPA: eicosapentaenoic acid; DHA: docosahexaenoic acid; OA: oleic acid; LA: linoleic acid; ALA: α-linolenic acid.

Supplementary Table 2. Correlations with statistical significance between different growth parameters and the expression of genes in liver of gilthead seabream fed the experimental diets. The correlation was considered significant at the bilateral levels of 0.05 (*) or 0.01 (**).

|  | **Liver *elovl5*** | **Liver *igf2*** | **Lipid Intake** | **Iodine Intake** | **Selenium Intake** |
| --- | --- | --- | --- | --- | --- |
| **FBW** | -0.636* | - | - | -0.755** | -0.755** |
| **WG** | -0.776** | - | 0.853** | - | -0.622* |
| **DGI** | -0.713** | - | 0.755** | - | - |
| **FCR** | 0.720** | - | -0.727** | - | 0.685* |
| **SGR** | -0.692* | - | 0.734** | - | - |
| **PER** | -0.713* | - | 0.713** | -0.720** | -0.699* |
| **Lipid Intake** | -0.692* | - | - | - | - |
| **Protein Retention** | -0.685* | 0.713* | - | - | - |
| **Protein Gain** | -0.741** | 0.638* | 0.594* | - | - |
| **Liver *selk*** | - | 0.447* | - | - | - |
| **Liver *dio2*** | - | 0.393* | - | - | - |
